# *Peptoniphilus coli* sp. nov. and *Peptoniphilus urinae* sp. nov., isolated from humans

**DOI:** 10.1007/s00203-022-03044-z

**Published:** 2022-07-20

**Authors:** Babacar Mbaye, Cheikh Ibrahima Lo, Niokhor Dione, Sarah Benabdelkader, Maryam Tidjani Alou, Samy Brahimi, Nicholas Armstrong, Stéphane Alibar, Didier Raoult, Valérie Moal, Matthieu Million, Pierre-Edouard Fournier, Florence Fenollar

**Affiliations:** 1 Aix Marseille Univ, IRD, AP-HM, MEPHI, Marseille, France; 2 IHU-Méditerranée Infection, Institut Hospitalo-Universitaire Méditerranée-Infection, 19-21 Boulevard Jean Moulin, 13385 Marseille cedex 05, France; 3 Aix Marseille Univ, IRD, AP-HM, SSA, VITROME, Marseille, France; 4 AP-HM, Hôpital Conception, Centre de Néphrologie Et Transplantation Rénale, Centre Hospitalo-Universitaire Conception, Marseille, France

**Keywords:** *Peptoniphilus coli* sp. nov., *Peptoniphilus urinae*, Taxonogenomics, Kidney transplant, Human microbiota, Bacteria

## Abstract

Strains Marseille-P3761 and Marseille-P3195 are representatives of two bacterial species isolated from human specimens. Strain Marseille-P3761 was isolated from the stool of a healthy volunteer, while strain Marseille-P3915 was cultivated from the urine of a kidney transplant recipient. Both strains are anaerobic Gram-positive coccoid bacteria. Both are catalase-negative and oxidase-negative and grow optimally at 37 °C in anaerobic conditions. They also metabolize carbohydrates, such as galactose, glucose, fructose, and glycerol. The major fatty acids were hexadecanoic acid for both strains. The highest digital DNA–DNA hybridization (dDDH) values of Marseille-P3761 and Marseille-P3195 strains when compared to their closest phylogenetic relatives were 52.3% and 56.4%, respectively. Strains Marseille-P3761 and Marseille-P3195 shared an OrthoANI value of 83.5% which was the highest value found with *Peptoniphilus* species studied here. The morphological, biochemical, phenotypic and genomic characteristics strongly support that these strains are new members of the *Peptoniphilus* genus. Thus, we suggest that *Peptoniphilus coli* sp. nov., and *Peptoniphilus urinae* sp. nov., are new species for which strains Marseille-P3761 (CSUR P3761 = CCUG 71,569) and Marseille-P3195 (CSUR P3195 = DSM 103,468) are their type strains, respectively of two new *Peptoniphilus* species, for which we propose the names *Peptoniphilus coli* sp. nov. and *Peptoniphilus urinae* sp. nov., respectively.

**Supplementary Information:**

The online version contains supplementary material available at 10.1007/s00203-022-03044-z.

## Introduction

Understanding the role that bacterial diversity plays in diseases that affect humans is essential (Turnbaugh et al. 2007). The implementation of the Culturomics strategy, a concept which is based on diversified culture conditions to enlarge our knowledge of the human microbiota, has enabled the discovery of many previously uncultured bacteria (Lagier et al. 2016, 2018). To describe new bacterial isolates, we used the taxono-genomics approach that includes matrix-assisted laser desorption/ionization time-of-flight (MALDI-TOF) mass spectrometry (MS) analysis, phylogenetic inference, description of the main phenotypic characteristics and genome sequencing and comparison to describe them (Fournier and Drancourt 2015; Fournier et al. 2015).

The genus *Peptoniphilus* was described by Ezaki (2001). Members of this genus are Gram-positive anaerobic cocci, non-motile, non-saccharolytic, and their major energy source results from the use of peptones and oligopeptides (Song et al. 2007). *Peptoniphilus* spp. have mostly been isolated from human clinical samples (Ezaki et al. 2001; Citron et al. 2012; Diop et al. 2019) and in some rare cases they are isolated from animals like swine, cattle or pig (Rooney et al. 2011; Wylensek et al. 2020; Ryu et al. 2021). Currently, there are more than 20 species validly published with standing in the nomenclature (https://lpsn.dsmz.de/genus/peptoniphilus).

Herein, we report the description of two new *Peptoniphilus* species named *Peptoniphilus urinae* sp. nov., strain Marseille-P3195 (previously reported) (Brahimi et al. 2017) and *Peptoniphilus coli* sp. nov., strain Marseille-P3761, both of which were isolated from human samples.

## Materials and methods

### Strain isolation and identification

Strain Marseille-P3195 was isolated in a urine sample from a young man who had just received a kidney transplant for genetic focal segmental glomerulosclerosis (Brahimi et al. 2017), while strain Marseille-P3761 was isolated in a fresh stool sample of an obese female volunteer living in France. Individuals gave their free and informed consent for the project before sampling. The study was approved by the ethics committee of Institut Fédératif de Recherche IFR48 under number 2016-010 as part of a long-term culturomics study of the human microbiota. These two bacterial strains were retrieved seven days after pre-incubation in an anaerobic blood culture bottle (Becton-Dickinson Diagnostics, Le Pont-de-Claix, France) supplemented with 5% sheep blood at 37 °C.

Attempts were made to identify bacterial colonies using MALDI-TOF MS (Bruker Daltonics, Bremen, Germany), as previously reported (Lo et al. 2015). The obtained spectra were imported and analyzed using Biotyper 3.0 software. They were then incremented in our local mass spectrometry database (https://www.mediterranee-infection.com/urms-data-base).

No identification was successful using MALDI-TOF MS. Therefore, sequencing of the 16S rRNA gene was performed for each of the studied strains using the primer pair fD1 and rP2 (Weisburg et al. 1991). They were sequenced using the Big Dye^®^ Terminator v1.1 Cycle Sequencing Kit and 3500xL Genetic Analyzer capillary3500xL sequencer (Thermo Fisher, Saint-Aubin, France), as previously described (Lo et al. 2016). The 16S rRNA nucleotide sequences were assembled and corrected using CodonCode Aligner software (http://www.codoncode.com). Sequences were aligned using MUSCLE with default parameters, phylogenetic inference were obtained using the maximum likelihood method and the MEGA 7 software. Numbers at the nodes are percentages of bootstrap values obtained by repeating the analysis 1000 times to generate a majority consensus tree. The scale bar indicates a 1% nucleotide sequence.

### Phenotypic and biochemical characteristics

Bacterial colonies of strains Marseille-P3761 and Marseille-P3195 appear distinctly on 5% sheep blood-enriched Columbia agar (bioMérieux, Marcy l'Étoile, France). Phenotypic tests, such as Gram staining, sporulation, catalase and oxidase reactions, were performed for each strain, as previously reported (Brahimi et al. 2017). The optimal temperature and pH for the growth of each strain were sought. Strains were inoculated on 5% sheep blood-enriched Columbia agar (bioMérieux) and incubated under different temperatures (20, 28, 32, 37, 45, and 56 °C) and different pH levels (5, 6, 7, 7.5, 8, and 8.5). In addition, biochemical properties, such as carbohydrate metabolism and enzymatic activities, of these two strains were revealed using the API strips test (ZYM and 50 CH, bioMérieux). The morphology of the bacterial cells of each strain was highlighted using a TM4000 microscope (Hitachi Group, Krefeld, Germany). For each strain, 50 mg of bacterial biomass was collected from several culture plates to prepare the fatty acid methyl ester analysis, as previously described (Sasser 2006).

### Genomes sequencing and analysis

The total DNA of the genomes was recovered using the EZ1 biorobot (Qiagen, Courtaboeuf, France) and the EZ1 DNA tissue kit. Sequencing was performed using MiSeq technology (Illumina, San Diego, CA, USA) with the Nextera Mate Pair sample prep kit and Nextera XT paired end (Illumina), as previously described (Morel et al. 2015). Several bioinformatic tools, including Velvet (Zerbino and Birney 2008), Spades (Bankevich et al. 2012), and SOAPdenovo (Luo et al. 2012) were used to assemble the genomes. GapCloser software (Xu et al. 2019) was used to reduce assembly gaps. Scaffolds which had fewer than 800 base pairs (bp) or had a depth value lower than 25% of the mean depth were removed. The best assembly was selected using different criteria (number of scaffolds, N50, number of N). The degree of genomic similarity of each strain was evaluated by processing sequences using the Orthologous ANI Tool (OAT) software (Lee et al. 2016). Furthermore, the Genome-to-Genome Distance Calculator (GGDC) web server, which is available online (http://ggdc.dsmz.de), was used to calculate digital DNA–DNA hybridisation (dDDH) values between the genomes of closest species, as previously described (Meier-Kolthoff et al. 2013).

## Results and discussion

### Growth conditions of strains

Different growth temperatures and pH levels were tested with these strains. They all grew optimally at 37 °C in anaerobic conditions. The optimal pH was 7 for strain Marseille-P3761 and Marseille-P3195. Bacterial strains grew well, with distinct colonies on 5% sheep blood-enriched Columbia agar.

### Morphology and biochemical characteristics

The colonies of the two bacterial strains are similar; they appear gray and circular on 5% sheep blood-enriched Columbia agar. The two strains are Gram-positive cocci and catalase-negative. All biochemical properties, such as enzymatic activities and carbohydrates fermentation, for these two strains are revealed using API ZYM and API 50 CH, respectively. The reactions observed with API strips tests are reported in the Supplementary Table S1.

The main biochemical characteristics of these strains were compared with those of other closely related *Peptoniphilus* species (Table 1). The cell morphology of each strain was revealed by scanning electron microscope. They are sphere-shaped bacteria and can aggregate in duplicate (Supplementary Figure S1). Hexadecanoic acid was detected as a major fatty acid for strains Marseille-P3761 (38%) and Marseille-P3195 (31%). Minor amounts of unsaturated and other saturated structures were also detected (Supplementary Table S2).

**Table 1:** Comparison of differential characteristics of *Peptoniphilus coli* Marseille-P3761, *Peptoniphilus urinae* Marseille-P3195, *Peptoniphilus coxii* CCUG 59622^T^*, Peptoniphilus obesi* ph1^T^, and *Peptoniphilus asaccharolyticus* DSM 20463^T^

Properties	*P. coli*	*P. urinae*	*P. coxii*	*P. obesi*	*P. asaccharolyticus*
Cell diameter (μm)	0.5	0.8	0.7	0.7–0.9	na
Oxygen requirement	−	−	−	−	−
Gram stain	+	+	+	+	+
Salt requirement	−	−	−	−	−
Motility	−	−	−	−	−
Endospore formation	−	−	−	−	−
Alkaline phosphatase	−	−	−	−	−
Catalase	−	−	−	−	−
Oxidase	−	−	−	−	−
β-galactosidase	−	−	−	−	−
N-acetyl-glucosamine	−	−	−	−	na
Arabinose	−	−	−	−	na
Lipase (C8)	−	w	na	−	na
Mannose	−	−	+	+	−
Mannitol	+	−	na	na	na
Sucrose	+	−	na	−	−
D-glucose	+	−	na	na	−
D-fructose	+	−	na	na	−
D-maltose	+	−	na	na	−
Source	Human	Human	Human	Human	Human

*+* Positive, *−* negative, *w* weak, *na* no available data

### Phylogenetic identification

**Fig. 1:**
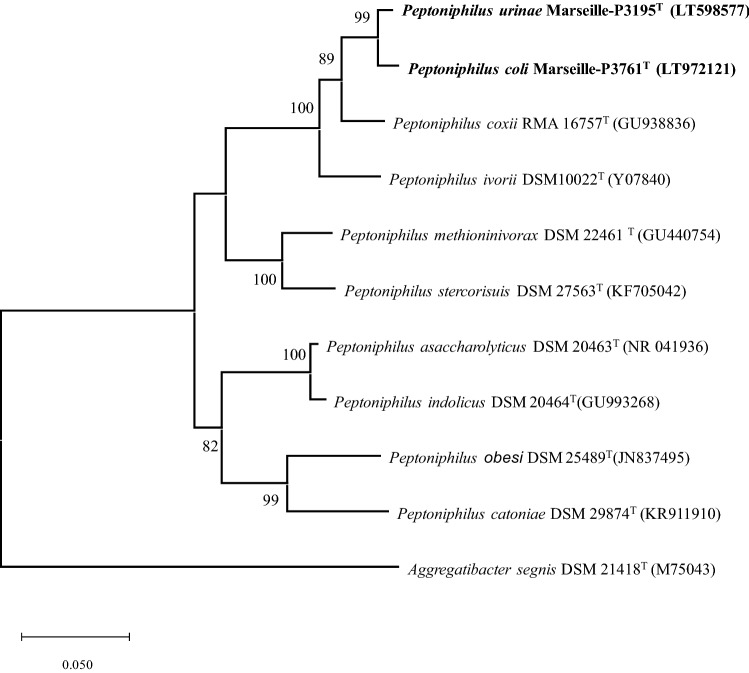
Phylogenetic trees highlighting the position of *Peptoniphilus coli* sp. nov. and *Peptoniphilus urinae* sp. nov., based on the 16S rRNA gene sequences relative to the most closely related type strains within the genus *Peptoniphilus*. Genbank accession numbers are indicated in parentheses

The Blastn against the 16S rRNA gene sequence GenBank database revealed that strain Marseille-P3761 and strain Marseille-P3195 both exhibited 95.7% and 96.0% sequence identity with *Peptoniphilus coxii* RMA 16757 (GenBank accession number NR_117556.1). Furthermore, strains Marseille-P3761 (LT972121) and Marseille-P3195 (LT598577) had 98.46% sequence similarity of the 16S rRNA gene out of 97% coverage. The values obtained were below the threshold value of 98.65% recommended to delimit new prokaryotic species (Kim et al. 2019). Given these phylogenetic criteria, we consequently classified these strains as new members belonging to the genus *Peptoniphilus* within the phylum *Firmicutes*. In addition, the phylogenetic tree (Fig. 1) shows the positions of these two new species among related *Peptoniphilus* species with a validly published name.

**Fig. 2:**
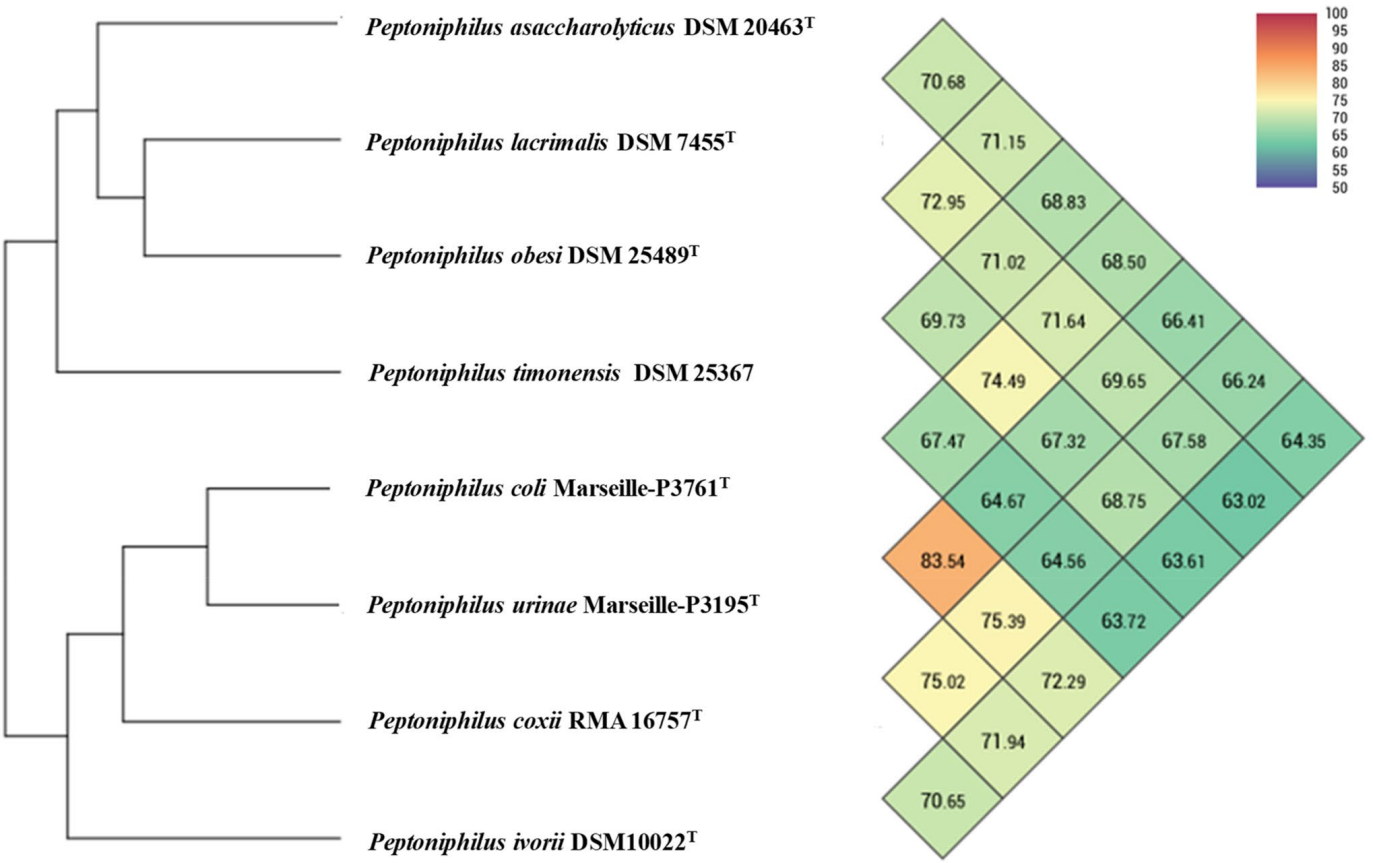
Heatmap generated with OrthoANI values calculated using the OAT software between *Peptoniphilus coli* Marseille-P3195 and *Peptoniphilus urinae* Marseille-P3761and other closely related species with standing in nomenclature

### Comparison and genomic specificities

The genome size of *Peptoniphilus coli* strain Marseille-P3761 was 1,986,843 bp long with a 48.6 mol% G + C content, whereas the genome of *Peptoniphilus urinae* strain Marseille-P3195, was 1,822,830 bp with a 49.7 mol% G + C content (Supplementary Figure S2).

The dDDH values ranged from 17.5% between *Peptoniphilus ivorii* DSM 10022^T^ and *Peptoniphilus coxii* CCUG 59622^T^*,* to 60.2% between *P. coli* and *P. obesi* ph1^T^. Strains Marseille-P3761 and Marseille-P3195 had the highest dDDH values of 60.2% and 52.4%, respectively, in this analysis (Table 2).

**Table 2:** Genomic comparison of *Peptoniphilus coli* Marseille-P3761^T^ and *Peptoniphilus urinae* Marseille-P3195^T^ between their closely related species (*Peptoniphilus obesi* ph1^T^, *Peptoniphilus lacrimalis* GIFU 7667^T^, *Peptoniphilus timonensis* CSUR P165^T^, *Peptoniphilus coxii* CCUG 59622^T^, *Peptoniphilus asaccharolyticus* DSM 20463^T^, and *Peptoniphilus ivorii* DSM 10022^T^) using GGDC and formula 2 (dDDH estimates based on identities over HSP length)

	Marseille-P3195^T^	Marseille-P3761^T^	GIFU 7667^ T^	CSUR P165^T^	CCUG 59622^ T^	DSM 20463^ T^	DSM 10022^ T^	ph1^T^
Marseille-P3195^T^	100%	27.2 ± 2.4%	56.4 ± 2.7%	34.3 ± 2.4%	20.1 ± 2.3%	32.3 ± 2.4%	18.0 ± 2.2%	39.8 ± 2.5%
Marseille-P3761^T^		100%	52.3 ± 2.6%	51.1 ± 2.6%	20.4 ± 2.3%	45.1 ± 2.6%	18.1 ± 2.2%	60.2 ± 2.8%
GIFU 7667^ T^			100%	21.9 ± 2.3%	40.6 ± 2.5%	31.9 ± 2.4%	30.9 ± 2.4%	32.3 ± 2.4%
CSUR P165^T^				100%	27.3 ± 2.4%	27.2 ± 2.4%	33.7 ± 2.4%	25.2 ± 2.4%
CCUG 59622^ T^					100%	35.4 ± 2.4%	17.5 ± 2.2%	40.4 ± 2.5%
DSM 20463^ T^						100%	35.4 ± 2.5%	27.7 ± 2.4%
DSM 10022^ T^							100%	37.5 ± 2.5%
ph1^T^								100%

OrthoANI values among closely related species (Fig. 2) ranged from 83.5% between *Peptoniphilus coli* and *Peptoniphilus urinae* to 63% between *Peptoniphilus lacrimalis* and *Peptoniphilus ivorii*. However, *P. coli* had higher genomic similarity with *P. urinae* (83.5%). *P. coli* and *P. urinae* had lower values with *P. timonensis*, ranging from 67.4% to 64.6%, respectively.

COG analysis showed that genes encoding extracellular structures, prophages, transposons, and general function prediction only were most present in the genomes of strains Marseille-P3761 and Marseille-P3195. The distribution of genes in the 25 general COG categories is illustrated in Supplementary Figure S2.

## Conclusion

Based on the results from unique phenotypic criteria, MALDI-TOF spectra, phylogenetic and genomic characteristics, such as 16S rRNA sequence similarity lower than 98.65% and OrthoANI value lower than 95%, with the phylogenetically closest species with standing in the nomenclature, we formally proposed strain Marseille-P3761^T^ and Marseille-P3195^T^ as respectively the type strains of *Peptoniphilus coli* sp. nov., and *Peptoniphilus urinae* sp. nov.

## Description of *Peptoniphilus coli* sp. nov.

*Peptoniphilus coli* (co’li. L. gen. n. coli, of the colon). Colonies appear gray and circular. Cells are Gram-positive sphere-shaped, non-spore forming. Catalase and oxidase activities are not detected. Optimal growth is obtained at 37 °C in anaerobic atmosphere on 5% sheep blood-enriched Columbia agar.

It is a Gram-positive sphere-shaped bacterium with a mean length of 1.2 μm and a mean diameter of 0.5 μm.

It exhibits positive reactions for esterase (C4), α-chymotrypsin, naphthol-AS-BI-phosphohydrolase, glycerol, galactose, glucose, fructose, mannitol, esculin ferric citrate, trehalose and D-turanose. The major fatty acids are C_16:0_ (38%) and C_18:1n9_ (30%). The genome size and G + C content are 1,986,843 bp and 48.6 mol%, respectively.

The type strain Marseille-P3761^T^ (= CSUR P3761^T^ = CCUG 71569^T^) was isolated in a stool sample from a healthy French volunteer.

The genome and 16S rRNA gene sequence were deposited in GenBank under accession numbers OPYI00000000 and LT972121, respectively.

## Description of *Peptoniphilus urinae* sp. nov.

*Peptoniphilus urinae* (u.ri’nae. L. gen. n. *urinae*, of urine). Colonies are gray and circular. Bacterial cells are Gram-positive sphere-shaped, non-spore forming. Catalase or oxidase activities are not detected. Optimal growth is at 37 °C in anaerobic atmosphere on sheep blood-enriched Columbia agar. It exhibits positive reactions for glycerol, fucose, raffinose, maltose, lactose, melibiose, *D*-trehalose, esculin ferric citrate, *N*-acetyl-glucosamine, sorbitol, dulcitol, galactose, glucose, fructose, arabinose, ribose, xylose, esterase lipase (C8), acid phosphatase, and naphthol-AS-BI-phosphohydrolase. The major fatty acids are C_16:0_ (31%), C_18:1n9_ (25%), and C_18:2n6_ (24.2%). The genome size and G + C content are 1,822,830 bp and 49.7 mol%, respectively.

The type strain Marseille-P3195^T^ (= CSUR P3195^T^ = DSM 103468^T^) was isolated in a urine sample from a young man who had just received a kidney transplant for genetic focal segmental glomerulosclerosis.

The genome and 16S rRNA sequence were deposited in GenBank under accession numbers FTPC00000000 and LT598577, respectively.

## Supplementary Information

Below is the link to the electronic supplementary material.

Supplementary file1 (DOCX 423 KB)
