# Correction to: *Peptoniphilus coli* sp. nov. and *Peptoniphilus urinae* sp. nov., isolated from humans

**DOI:** 10.1007/s00203-022-03248-3

**Published:** 2022-09-27

**Authors:** Babacar Mbaye, Cheikh Ibrahima Lo, Niokhor Dione, Sarah Benabdelkader, Maryam Tidjani Alou, Samy Brahimi, Nicholas Armstrong, Stéphane Alibar, Didier Raoult, Valérie Moal, Matthieu Million, Pierre-Edouard Fournier, Florence Fenollar

**Affiliations:** 1 Aix Marseille Univ, IRD, AP-HM, MEPHI, Marseille, France; 2 IHU-Méditerranée Infection, Institut Hospitalo-Universitaire Méditerranée-Infection, 19-21 Boulevard Jean Moulin, 13385 Marseille cedex 05, France; 3 Aix Marseille Univ, IRD, AP-HM, SSA, VITROME, Marseille, France; 4 Centre de Néphrologie Et Transplantation Rénale, AP-HM, Hôpital Conception, Centre Hospitalo-Universitaire Conception, Marseille, France

## Correction to: Archives of Microbiology (2022) 204:506 10.1007/s00203-022-03044-z

The authors regret that they forgot to add the correct supplementary Table S2 in the supplementary data section. The correct supplementary Table S2 corresponds to unsaturated and saturated fatty acids of strains Marseille-P3761T and Marseille-P3195T instead of the phenotypic characteristics of the Marseille-P3761 and Marseille-P3195 strains obtained by using API ZYM. The phenotypic characteristics obtained by using API ZYM should be presented with those of API 50CH in Supplementary Table S1. The authors would like to apologize for the inconvenience caused.

## Supplementary Information

Below is the link to the electronic supplementary material.

Supplementary file1 (DOCX 426 KB)

