# Supplementary material for: Correction to: Peptoniphilus coli sp. nov. and Peptoniphilus urinae sp. nov., isolated from humans
Source: Arch Microbiol. 2022 Sep 27;204(10):645. doi: 10.1007/s00203-022-03248-3 (PMC9515011; doi:10.1007/s00203-022-03248-3)
Supplement: Supplementary file 1 — Supplementary file1 (DOCX 426 KB) [file 203_2022_3248_MOESM1_ESM.docx]

**Supplementary Table S1**: Phenotypic characteristics of strains Marseille-P3761 and Marseille-P3195.

**A. Phenotypic characteristics obtained by using API 50 CH.**

| **Characteristics** | **Marseille-P3761^T^** | **Marseille-P3195^T^** |
| --- | --- | --- |
| Glycerol | + | + |
| Erythritol | w | w |
| D-arabinose | - | + |
| L-arabinose | - | + |
| D-ribose | - | + |
| D-xylose | - | + |
| L-xylose | - | w |
| D-Adonitol | - | - |
| Methyl βD-xylopyranoside | - | w |
| D-galactose | + | + |
| D-glucose | + | + |
| D-fructose | + | + |
| D-mannose | - | + |
| L-sorbose | w | - |
| L-rhamnose | - | + |
| Dulcitol | - | + |
| Inositol | - | - |
| D-mannitol | + | + |
| D-sorbitol | - | + |
| Methyl αD-mannopyranoside | - | w |
| Methyl αD-glucopyranoside | - | w |
| N-acetyl-glucosamine | w | + |
| Amygdalin | - | - |
| Arbutin | - | + |
| Esculin ferric citrate | + | + |
| Salicin | - | w |
| D-cellobiose | - | w |
| D-maltose | + | + |
| D-lactose | + | + |
| D-melibiose | - | + |
| D-saccharose | + | + |
| D-trehalose | + | + |
| Inulin | - | w |
| D-melezitose | + | - |
| D-raffinose | - | + |
| Amidon | - | w |
| Glycogen | - | - |
| Xylitol | w | w |
| Gentiobiose | w | - |
| D-turanose | + | - |
| D-xylose | - | - |
| D-tagalose | - | w |
| D-fucose | - | - |
| L-fucose | - | + |
| D-arabitol | - | w |
| L-arabitol | - | w |
| Potassium gluconate | - | + |
| Potassium 2-ketogluconate | - | - |
| Potassium 5-ketogluconate | - | w |

**B. Phenotypic characteristics obtained by using API ZYM.**

| **Characteristics** | **P3761^T^** | **P3195^T^** |
| --- | --- | --- |
| Alkaline phosphatase | w | w |
| Esterase (C4) | + | + |
| Esterase Lipase (C8) | - | + |
| Lipase (C14) | - | - |
| Leucine arylamidase | - | w |
| Valine arylamidase | - | - |
| Cystine arylamidase | - | + |
| Trypsin | w | w |
| α-chymotrypsin | + | - |
| Acid phosphatase | - | + |
| Naphthol-AS-BI-phosphohydrolase | + | + |
| α-galactosidase | - | - |
| β-galactosidase | - | - |
| β-glucuronidase | - | - |
| α-glucosidase | - | - |
| β-glucosidase | - | - |
| N-acetyl-β-glucosaminidase | - | - |
| α-mannosidase | - | - |
| α-fucosidase | - | - |

**Supplementary Table S2**: Unsaturated and saturated fatty acids of strains Marseille-P3761^T^ and Marseille-P3195^T^.

| **Fatty acids** | **Name** | **Marseille-P3761^T^** | **Marseille-P2932^T^** |
| --- | --- | --- | --- |
| C_16:00_ | Hexadecanoic acid | 38.0 | 27.7 |
| C_18:00_ | Octadecanoic acid | 4.1 | 3.5 |
| C_18:1n9_ | 9-Octadecenoic acid | 30.0 | 5.2 |
| C_14:00_ | Tetradecanoic acid | 8.2 | 44.3 |
| C_18:2n6_ | 9,12-Octadecadienoic acid | 9.7 | 1.9 |
| C_10:00_ | Decanoic acid | 1.6 | 1.0 |
| C_12:00_ | Dodecanoic acid | TR | 10.9 |
| C_15:00_ | Pentadecanoic acid | TR | 2.1 |


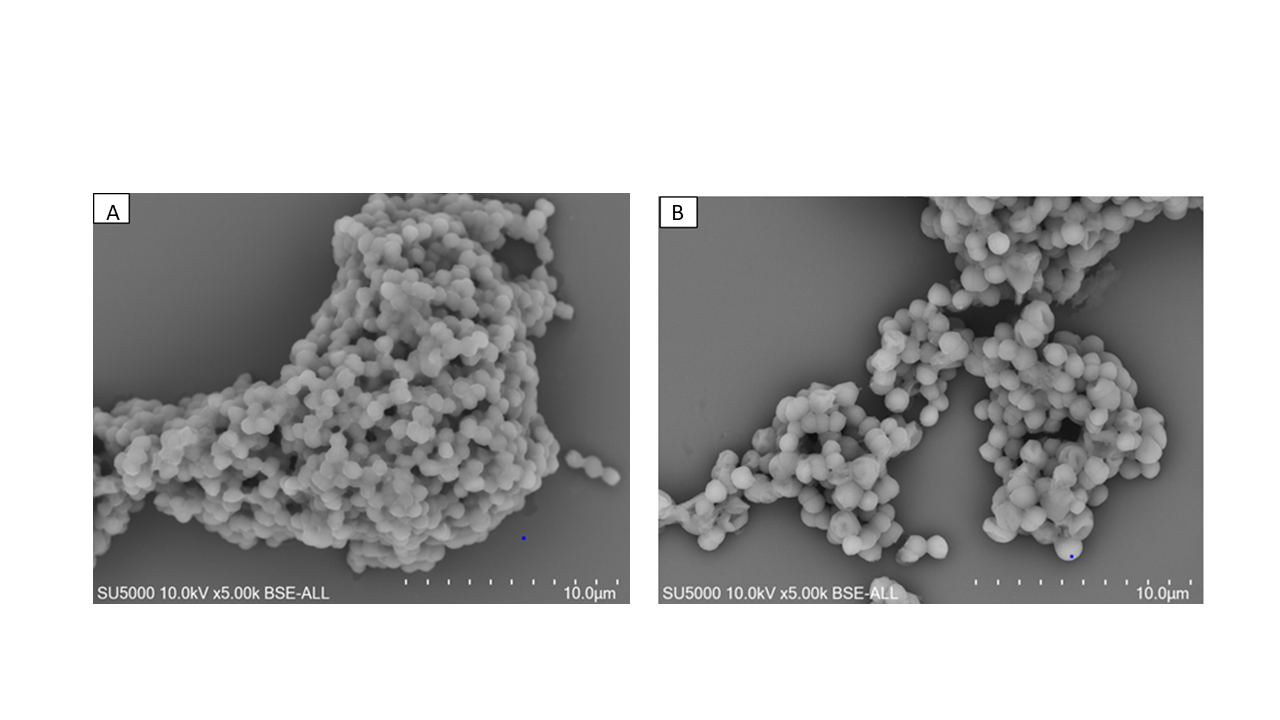


**Supplementary Figure S1**: Scanning electron microscopy of stained strain Peptoniphilus urinae sp. nov. Marseille-P3195^T^ (A) and Peptoniphilus coli sp. nov. Marseille-P3761^T^ (B).

**Supplementary Figure S2**: Distribution of functional classes of predicted genes according to the COG of proteins. **1:** Peptoniphilus ivorii, **2:** Peptoniphilus asaccharolyticus, **3:** Peptoniphilus urinae sp. nov., **4:** Peptoniphilus timonensis, **5:** Peptoniphilus obesi, **6:** Peptoniphilus lacrimalis, **7:** Peptoniphilus coxii, **8:** Peptoniphilus coli sp. nov.
